# Dual Role of Beam Polarization and Power in Nematic Liquid Crystals: A Comprehensive Study of TE- and TM-Beam Interactions

**DOI:** 10.3390/ma17050999

**Published:** 2024-02-22

**Authors:** Michał Kwaśny, Bartłomiej Wojciech Klus, Urszula Anna Laudyn

**Affiliations:** Faculty of Physics, Warsaw University of Technology, Koszykowa 75, 00-662 Warsaw, Poland; michal.kwasny@pw.edu.pl (M.K.); bartlomiej.klus@pw.edu.pl (B.W.K.)

**Keywords:** spatial solitons, reorienational nonlinearity, thermal nonlinearity, diametric drive

## Abstract

Optical spatial solitons are self-guided wave packets that maintain their transverse profile due to the self-focusing effect of light. In nematic liquid crystals (NLC), such light beams, called nematicons, can be induced by two principal mechanisms: light-induced reorientation of the elongated molecules and thermal changes in the refractive index caused by partial light absorption. This paper presents a detailed investigation of the propagation dynamics of light beams in nematic liquid crystals (NLCs) doped with Sudan Blue dye. Building on the foundational understanding of reorientational and thermal solitons in NLCs and the effective breaking of the action–reaction principle in spatial solitons, this study examines the interaction of infrared (IR) and visible beams in a [-4-(trans-4′-exylcyclohexyl)isothiocyanatobenzene] (6CHBT) NLC. Our experimental results highlight the intricate interplay of beam polarizations, power levels, and the nonlinear properties of NLCs, offering new insights into photonics and nonlinear optics in liquid crystals.

## 1. Introduction

Exploring light–matter interactions in nematic liquid crystals (NLCs) has become a cornerstone of contemporary photonics research, unveiling phenomena with profound theoretical and practical implications. NLCs, characterized by their unique anisotropic optical properties, serve as a versatile medium for studying various nonlinear optical effects, including the formation and dynamics of spatial solitons called nematicons [1,2]. This research area bridges fundamental physics and technological innovations, leading to advancements in optical communication, signal processing, and material science.

The intriguing aspect of NLCs lies in their ability to manipulate light propagation through their molecular orientation, which can be controlled by external fields, temperature gradients, and light itself [3,4,5,6]. By inducing dipoles in the NLC molecules, the extraordinary component of the optical field is responsible for the rotation of the molecular optical axis, an effect called reorientational nonlinearity [3]. In addition, the NLCs exhibit additional substantial nonlinear mechanisms, i.e., thermal nonlinearity, associated with wavelength-dependent partial absorption. This responsiveness enables the study of various nonlinear optical effects, most notably the formation and propagation of solitons. Solitons in NLCs, specifically reorientational and thermal solitons, represent a crucial area of research due to their distinct characteristics and potential for practical applications [7,8,9,10].

Reorientational solitons, or “nematicons”, arise from the interplay between the light-induced reorientation of NLC molecules and the elastic restoring forces, creating self-trapped light beams [1,2]. On the other hand, thermal solitons emerge from localized temperature changes induced by light absorption, altering the refractive-index profile of the NLC [11,12,13,14]. These solitons offer a different perspective on light–matter interactions, where thermal effects play a pivotal role. Understanding thermal solitons and their impact on molecular reorientation is crucial for developing temperature-sensitive photonic devices and exploring the thermal dynamics in anisotropic materials. The coexistence and interplay between reorientational and thermal solitons in NLCs present a complex and affluent area of study. The response of NLCs to different light polarizations, such as transverse electric (TE) and transverse magnetic modes (TM), adds another layer of complexity. The coexistence of two competing nonlinear mechanisms, one of which (molecular reorientation) is always positive in sign and the second (i.e., the thermal effects) either negative or positive in sign, depending on the specific properties of the NLC, opens a wide range of different possibilities related to a non-trivial type of light–matter interaction in nonlinear reorientational soft matter [12,13,15,16]. A more detailed analysis of the nonlinearity coefficient versus temperature for the [-4-(trans-4′-exylcyclohexyl)isothiocyanatobenzene] (6CHBT) nematic liquid crystal [17] shows, among other things, the temperature ranges in which the two mechanisms leading to nonlinear refractive-index changes compete, coexist, or enhance each other [18]. This interplay, influenced by light intensity, beam polarization, and external fields, leads to a diverse range of nonlinear phenomena [7,18,19]. The differential interaction of these polarization states with NLCs has significant implications for the design of optical components, like beam steerers, modulators, and adaptive lenses. It also provides a deeper understanding of the anisotropic nature of these materials and their potential for creating tunable and adaptive optical systems.

Harnessing the competing and coexisting reorientational and thermal nonlinearities in suitably doped nematic liquid crystals (NLCs) presents significant advantages. These nonlinearities have been utilized to control soliton trajectories and positions within NLC cells. A particularly intriguing aspect is the mutual interaction of light beams in NLCs, leading to aligned accelerations, a phenomenon known as “diametric drive” [16,20,21,22]. This interaction, akin to two massive objects influencing each other in a manner that defies action–reaction symmetry, is an impossibility in classical mechanics but becomes feasible in the realm of NLCs, offering new frontiers in optical control and manipulation [23].

This paper aims to contribute to this growing field by presenting new insights into the dynamics of solitons in NLCs and exploring the implications of these findings for the future of photonics and advanced materials. We seek to expand the understanding of light propagation in anisotropic media and its potential applications in next-generation optical technologies through a combination of different experimental approaches.

## 2. Materials and Methods

In our study of nonlinear optics in NLCs, we introduce a guest dopant to the host LC mixture to heighten light absorption within a specific wavelength range. This allows for a detailed investigation into the interaction between reorientation, which typically dominates in pure NLCs, and thermo-optical effects. We explore these interactions by employing wavelengths inside and outside the dopant’s absorption band. Both nonlinearity mechanisms, i.e., reorientation and thermal ones, allow for a straightforward modification of the magnitude of the NLC nonlinear response. NLCs, as uniaxial materials, consist of elongated molecules aligned along the molecular director (optic axis) ***n***. The ordinary refractive index corresponds to eigenwaves with an electric field perpendicular to the ***n***; the extraordinary refractive index depends on the angle *θ* between the optical wave vector and ***n***. The value and spatial distribution of *θ* in an NLC defines the phase velocity and diffraction of a propagating light beam. The ordinary electric field (o-wave) is always perpendicular to the director ***n***, and its phase velocity is $c/n_{o}$. In contrast, the extraordinary electric field (*e*-wave) lies in the same plane as the wave vector ***k*** and the director ***n***, with a phase velocity of $c/n_{e}$, which depends on the orientation angle *θ* between ***k*** and ***n***. The refractive indices of NLCs are temperature-dependent, with n_e_ typically decreasing as the temperature increases and *n_o_* either increasing or decreasing depending on the material’s properties [3]. The exact relationship between temperature and refractive index depends on the specific NLC material and its temperature range.

The extraordinary wave polarization propagates in a θ-dependent refractive index: $n_{e}\left(\theta ,T\right)=\cos^{2}\theta /n_{o}^{2}(T)+\sin^{2}\theta /n_{e}^{2}{\left(T\right)}^{-\frac{1}{2}}$, with energy flux (Poynting vector) angularly displaced by the walk-off angle $\delta \left(\theta ,T\right)$ from the wave vector ***k***. This causes the beam trajectory to vary through changes in walk-off, the latter conveniently expressed as [24]

(1) $$\delta \left(\theta ,\;T\right)=\arctan\left[\frac{\epsilon_{a}\left(T\right)sin2\theta }{\epsilon_{a}\left(T\right)+2n_{o}^{2}\left(T\right)+\epsilon_{a}\left(T\right)cos2\theta }\right]$$

In the case of the mechanism based on reorientation, the magnitude of the nonlinear response, expressed by the *n_2R_* coefficient, depends both on the angle between the director and the electric component of the optical wave field and temperature. The nonlinear index change $\Delta n_{e}^{2}\left(\theta ,T\right)=n_{e}^{2}\left(\theta ,T\right)-n_{e}^{2}(\theta_{0},T)$ depends on the director distribution, where the optic axis distribution is given by $\theta =\theta_{0}+\psi$, i.e., the superposition of the reorientation at rest (*θ*_0_) and the nonlinear reorientation ψ due to the beam. The nonlinearity, hence the degree of confinement of the solitary beam, can be expressed as [7]

(2) $$n_{2R}\left(\theta_{0},T\right)=2\epsilon_{0}\;\left(\frac{\epsilon_{a}\left(T\right)}{K\left(T\right)}\right)\sin\left[2\left(\theta_{0}-\delta \left(\theta_{0},T\right)\right)\right]n_{e}^{2}\left(\theta_{0},\;T\right)tan\delta \left(\theta_{0},T\right),$$

where $\epsilon_{a}(T)=n_{e}^{2}(T)-n_{o}^{2}(T)$ is the optical anisotropy and K(T) the Frank elastic constant, quantifying the temperature-dependent strength of the intermolecular links.

In the tailored design of an NLC cell, the molecular alignment direction is strategically determined to achieve a specific level of nonlinear optical response. The absolute value of the nonlinear coefficient n_2T_, which is related to the thermal nonlinearity mechanism, depends on the temperature characteristics of the refractive indices for a given NLC compound. It can be expressed by $n_{2T}=\sigma \cdot dn/dT$, where σ and *n* are the absorption coefficient and the ordinary or extraordinary refractive index, respectively. By increasing the linear absorption of light, for example, by doping absorbent dyes, the magnitude of the *n_2T_* coefficient can be adjusted to the desired level.

In experimental investigation, we deployed dual-wavelength optics to probe the intricate nonlinearity in nematic liquid crystals (NLCs) doped with Sudan Blue II [12,25], which purely enhances the thermal nonlinear response. Employing a transverse electric (TE) infrared Nd:YAG laser at *λ* = 1064 nm—chosen for its minimal absorption by the dye —and a transverse magnetic (TM) visible beam at *λ* = 642 nm or *λ* = 532 nm, aligned with the dopant’s peak absorption, we dissected the complex dynamics of nematicon propagation.

The experimental investigation was performed in planar NLC cells consisting of two glass plates bonded together with a gap of *d* = 80 μm and the homogeneous orientation of the director at *θ*_0_ = 30° with respect to the *z*-axis (the *z*-axis coincides with the wave vector ***k***). The cell was filled with a 6CHBT nematic liquid crystal doped with a 0.05% weight concentration of Sudan Blue II dye, with an absorption peak localized at *λ* = 604 nm and significantly enhanced absorption at *λ* = 532 nm [25].

**Figure 1:**
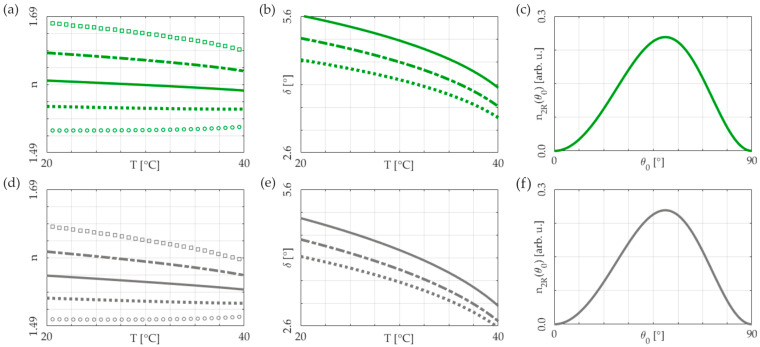
Optical and nonlinear parameters of 6CHBT nematic liquid crystal in the visible (**a**–**c**) wavelength spectral range as a function of temperature for different initial orientations: (**a**) refractive indices for various *θ*_0_ (circles *θ*_0_ = 0°, dotted line *θ*_0_ = 30°, solid line *θ*_0_ = 45°, dash-dotted line *θ*_0_ = 60°, and squares *θ*_0_ = 90°); (**b**) walk-off angle for initial molecular alignment at *θ*_0_ = 30°, *θ*_0_ = 45°, and *θ*_0_ = 60° (dotted, solid, and dash-dotted lines, respectively); (**c**) reorientational nonlinearity coefficient $n_{2R}(\theta_{0})$ at room temperature; (**d**–**f**) the same as in (**a**–**c**) for the infrared wavelength spectral range.

The initial orientation angle was chosen for two purposes: firstly, to induce a negative refractive gradient due to the temperature increase (manifesting as negative thermal nonlinearity for the *e*-wave) and, secondly, to balance the minimization of temperature sensitivity in the walk-off angle against the maximization of the reorientation nonlinearity coefficient *n*_2*R*_, as depicted in Figure 1.

Both input beams (orthogonally polarized, continuous wave (CW) characterized by a Gaussian electric-field profile) were collimated by a microscope objective to comparable widths in their waist (about 3 microns) and simultaneously coupled into the NLC medium. The beams remained close to each other and were aligned so that their Poynting vectors were kept parallel within the NLC cell. The light propagation was observed by the CCD camera combined with the microscope objective, which collected the out-of-plane scattered light in the *yz*-plane.

## 3. Experimental Results and Discussion

At this stage, our investigation centers on the TE-infrared-beam propagation, which operates through molecular reorientation and gives rise to a reorientational nematicon. This reorientation is contrasted against the effects of a temperature gradient produced by the highly absorbed visible TM-polarized wave on the NLC medium. Under the influence of the visible TM-polarized beam, the NLC’s ordinary refractive index increases, forming a red thermal nematicon. Simultaneously, this thermally induced soliton diminishes the extraordinary refractive index perceived by the TE-induced reorientation nematicon. According to (1) and Figure 1a,b, this temperature gradient introduces dual perturbations to the infrared beam’s trajectory: (1) a thermally induced modification of the walk-off angle due to birefringence changes and (2) a drift towards regions of higher refractive index. This, in turn, substantially alters the trajectory of the extraordinary beam. Please note that in this configuration, the TM-polarized beam does not perturb the optical parameters of the medium in any way that could affect the nature or the propagation direction of a TM-polarized beam. Despite the launch angle of the red transverse magnetic (TM) beam being slightly angled away from the *z*-axis direction, owing to the infrared-transverse-electric (TE)-beam walk-off angle, it encounters an ordinary refractive index when coupling, regardless of the coupling direction. In this way, for a TM-polarized beam, the NLC behaves as an isotropic medium with an index of refraction equal to the ordinary refractive index of the liquid crystal.

The nematicon trajectory’s alteration, consequent to the walk-off angle decreasing as a function of temperature, results in the propagation direction shifting toward the system’s origin. Furthermore, the increased temperature caused by the red beam reduces the refractive index encountered by the infrared beam, causing it to move away along the temperature gradient created. Depending on the specific geometry, i.e., whether the red beam is launched above or below the infrared beam, the cumulative or counteracting effect of these induced refractive-index gradients on the overall beam trajectory is critically essential, i.e., it can either complement or compete with the temperature-induced walk-off shift.

**Figure 2:**
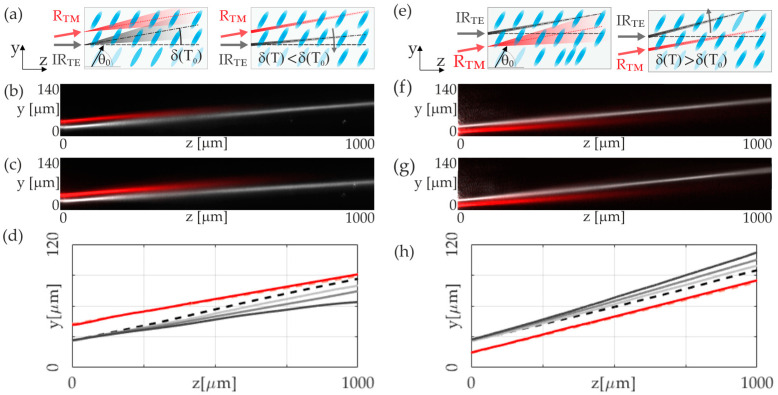
(**a**) Sketch of a liquid-crystal sample (6CHBT NLC doped with 0.05% of Sudan Blue dye, *θ*_0_ = 60°) with infrared (TE-polarized) and visible light (TM-polarized) beams coupling geometry and trajectories during propagation in NLC for a visible beam of low (left panel) and high (right panel) powers; (**b**,**c**) photographs of a simultaneous propagation in configuration form (**a**), presented for optical powers P_IR_ = 5.6 mW and P_RED_ = 0.4 mW, respectively; (**d**) trajectories of the infrared (P_IR_ = 5.6 mW) corresponding to increasing powers of a red beam, shown from light-to-dark grey solid lines, respectively: P_RED_ = 0.4 mW, P_RED_ = 2.7 mW, and P_RED_ = 4.0 mW. The dashed black line denotes the trajectory of the infrared beam in the case of independent propagation. Red dashed and solid lines refer to the visible beam of low and high powers; (**e**–**h**) refer to the same as in (**a**–**d**) for inverted-IR and visible-beam sequences.

Figure 2 provides a comprehensive illustration of these phenomena, depicting the NLC sample and the coupling geometry of the light beams and presenting experimental results. In Figure 2a, the top-view capture realized in the *yz*-plane shows a red beam launched above the infrared soliton. When a low-power red beam is used (P_RED_ = 0.4 mW), as presented in Figure 2b,c, the infrared soliton’s trajectory alteration is minimal, reducing the walk-off angle by about 0.4 degrees. However, increasing the power of the red beam up to 4 mW causes a further alteration in the infrared nematicon trajectory and a reduction in the effective walk-off angle by approximately 1.2 degrees, corresponding to an effective change in the *y*-coordinate of the soliton of 20 microns at a distance of *z* = 1000 μm. The exact trajectories of an infrared beam are plotted in Figure 2d. In the presented configuration, the thermal effects decrease the transverse shift in the nematicon, which resulted from the walk-off and light-induced molecular reorientation. The light-to-dark grey solid lines represent the trajectories of the TE beam along with the increased power of a highly absorbed red light beam. Simultaneously, we can see that the trajectory of the nematicon induced by thermal nonlinearity remains unaltered.

In the second configuration presented in Figure 2e, we indicate the red beam that is launched below the infrared nematicon. The experimental results are presented in Figure 2f,g. As in the low power configuration, the modification of the infrared soliton trajectory is small. The walk-off angle increases by 0.25 degrees, and the trajectory shifts towards a positive value on the *y*-axis. If the power of the red beam is increased, similar to the configuration in Figure 2a, the trajectory is further modified due to the repulsion of the infrared beam by the red beam. For a red beam power of 4 mW, the infrared soliton experiences a change in the walk-off angle of about one degree. This represents an increase in the walk-off angle relative to the propagation undisturbed by the red beam. The exact trajectories of an infrared beam are plotted in Figure 2h. Similar to Figure 2d, the light-to-dark grey solid lines represent the trajectories of the TE beam along with the increased power of a highly absorbed red light beam. The interaction changes the TE beam’s walk-off angle and propagation direction, highlighting the complexity of light–matter interactions within NLCs and the influence of polarization and temperature on soliton response. The detailed trajectory plots underscore the significance of beam placement and the refractive-index gradient’s role in steering the propagation within the medium.

**Figure 3:**
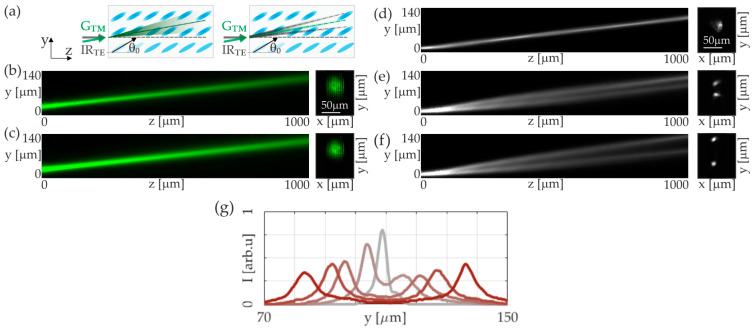
(**a**) Sketch of a liquid-crystal sample (6CHBT NLC doped with 0.05% of Sudan Blue dye, *θ*_0_ = 30°) with indicated coupling geometry of IR (TE-polarized) and visible light (TM-polarized) beams and their trajectories during propagation in the NLC: reported for low and high powers of TM-polarized light (left and right panels, respectively); (**b**,**c**) experimentally recorded mutual propagation of beams for P_IR_ = 6.5 mW, P_GREEN_ = 6.25 mW and P_IR_ = 6.5 mW, and P_GREEN_ = 6.5 mW: filtered view of a visible wavelength beam; (**d**) independent propagation of nematicon induced by an IR beam of a power P_IR_ = 6.5 mW and (**e**) co-propagation with a thermal nematicon induced by a visible beam of a power P_GREEN_ = 6.25 mW and (**f**) P_GREEN_ = 6.5 mW: filtered view of an IR spectral range. The right panels in (**b**–**f**) show the beam profile at the output of an NLC cell, at *z* = 1500 μm, filtered at particular wavelengths; (**g**) intensity distribution along the *y*-axis direction, at the propagation distance of *z* = 1000 μm, corresponding to (**f**), for increasing powers of the green beam: P_GREEN_ = 0 mW, 6.25 mW, 6.50 mW, 7.00 mW, and 7.50 mW (light-grey-to-dark-red lines, respectively).

The above results considered the effect of the thermal soliton on the propagation, particularly the direction of the reorientation soliton, and concerned configurations where the beams are introduced at some distance apart. The results presented in Figure 3 consider a configuration where both beams, the TE infrared and the visible TM, are launched along the same path. Similarly, as explained above, the TM beam with a power of P = 6.25 mW forms the thermal soliton, and the infrared TE beam forms the reorientational soliton. We show here that the interplay between both nonlinearities leads to extraordinary beam splitting and its propagation in the form of two solitons propagating at an angle dependent on the power of the ordinary beam (thermal soliton).

The power of the reorientational soliton is p = 6.5 mW. At this power, two breaths [19,26,27] were observed along the propagation path, indicating strong beam localization. Virtually no change in soliton propagation is observed when a green beam of initially low power is introduced along the same path. Both beams propagate without mutual influence. Increasing the power of the green beam generates a thermal soliton and thus reduces the extraordinary refractive index observed in the TE beam. The mechanism of the effect of the thermal soliton on the infrared beam is analogous to that described above: the temperature increase induced by the green beam reduces the extraordinary refractive index seen by the TE beam and modifies the value of the walk-off angle. This temperature increase causes the repulsion of the orientation soliton. In this case, the beams have been guided along a single path. This leads to the separation of the infrared beam and its further propagation in the form of two solitons, each with a lower power. The soliton is split into two for a thermal soliton power of p = 6.25 mW. A further increase in the thermal soliton power leads to the repulsion of these solitons, in agreement with the results presented earlier.

The results presented above detail the propagation of two orthogonally polarized beams with different wavelengths, where one wavelength is highly absorbed by the medium, causing a notable temperature increase. In contrast, the other is unaffected by the dye. Additionally, investigations were conducted with two beams of the same wavelength, specifically *λ* = 532 nm, within the dye’s enhanced absorption spectrum. Both TE- and TM-polarized beams at this wavelength lead to a rise in temperature during propagation. The study involved introducing TM and TE beams into a liquid-crystal cell separately and analyzing the nematicon formation and propagation. TM beams generate solitons through thermal nonlinearity, increasing the ordinary refractive index, whereas TE beams, through reorientation nonlinearity, cause an increase in the extraordinary refractive index and molecular rotation, leading to beam localization. The TE beam’s induced localization, accompanied by medium heating, results in a reduced effective refractive index and an altered walk-off angle, impacting the beam’s guidance direction. This effect on localization is observed primarily in the change in the guidance direction due to the walk-off angle shift.

In the next step, we study the independent propagation of beams at the wavelength of enhanced absorption of Sudan Blue II dye (*λ* = 532 nm). At this optical frequency, the thermal nonlinearity supports beam self-focusing for TM polarization in the form of a thermal soliton. On the other hand, the negative contribution of thermal effects (defocusing nonlinearity) for TE-polarized light is much weaker than the local increase in refractive index imposed by molecular reorientation. Thus, the stable propagation of a solitary wave is possible due to the reorientation nonlinearity mechanism for this particular polarization of light. Still, as the light absorption caused the temperature increase in the NLC, we observed a significant variation in the beam trajectory related to decreased birefringence. The defocusing character of the thermal nonlinearity for the extraordinary component is compensated by the reorientational nonlinearity, thus ensuring the formation of self-collimated beams in both polarizations.

**Figure 4:**
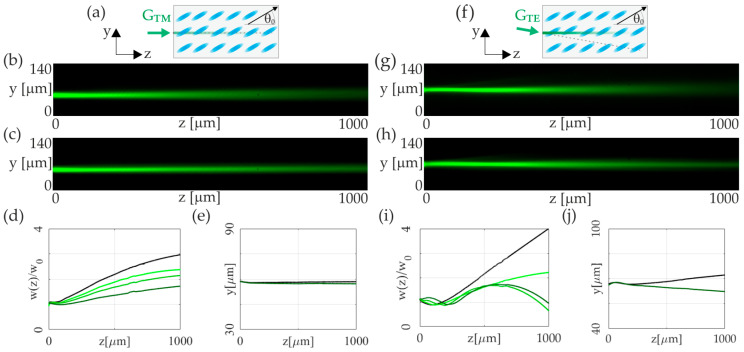
Analysis of unperturbed Gaussian beam propagation (*λ* = 532 nm) in 6CHBT NLC doped with 0.05% of Sudan Blue dye: (**a**) sketch of the NLC cell (*θ*_0_ = 30°) and the geometry of the TM-polarized-light-beam coupling; (**b**,**c**) the *yz*-plane view photographs of the TM-beam propagation for the optical powers of P_TM_ = 1.0 mW and P_TM_ = 6.0 mW, respectively; (**d**,**e**) evolution of the beam width and trajectory versus propagation distance, plotted for optical powers P_TM_ = 1.0 mW (black line) and 4.0 mW, 5.0 mW, and 6.0 mW (light-to-dark green lines, respectively); (**f**) the sketch of the NLC cell (*θ*_0_ = 30°) and the geometry of the TE-polarized-light-beam coupling; (**g**–**j**) the same as in (**b**–**e**) regarding the TE-polarized-light-beam propagation in the configuration shown in (**f**).

Figure 4 illustrates the evolution of a TM-and-TE green light beam in a homogeneously aligned NLC cell. The TM-ordinary-wave propagation was observed in both low-power (P_GREEN_ = 0.5 mW) and high-power (P_GREEN_ = 6.0 mW) regimes, as shown in Figure 4b,c. An increase in optical power leads to a significant decrease in beam width without altering its trajectory, as depicted in Figure 4d,e, across various power levels from 1.0 mW to 6.0 mW.

The TE-polarized beam of the same wavelength was analyzed in the same NLC cell with a slightly tilted coupling direction to maintain propagation along the *z*-axis (Figure 4f). Low- and high-power regimes (P_GREEN_ = 0.5 mW and 5.0 mW, respectively) are presented in Figure 4g,h. The TE beam, supporting molecular reorientation, starts forming a solitary wave at P_GREEN_ = 4.0 mW in Figure 4i. The thermal nonlinearity becomes more significant for higher powers, leading to notable walk-off variations. Comparing beam trajectory at P_GREEN_ = 0.5 mW and P_GREEN_ = 5.0 mW over *z* = 1000 μm reveals a transverse shift of approximately 10 μm, as demonstrated in Figure 4j.

**Figure 5:**
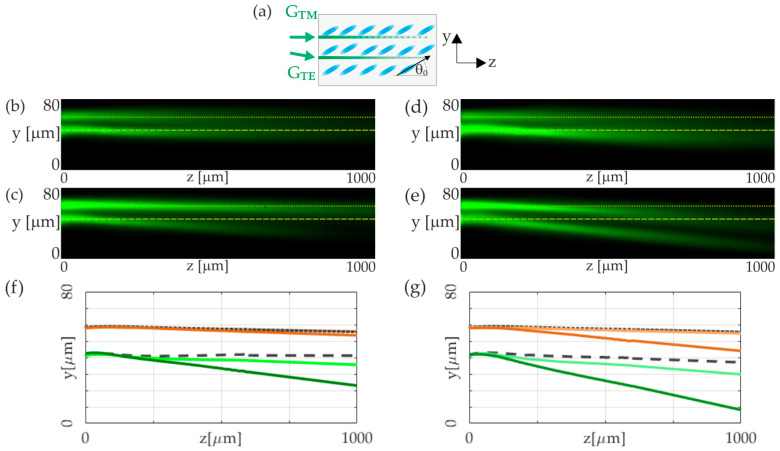
Co-propagation of TM- and TE-polarized Gaussian beams (*λ* = 532 nm) in 6CHBT NLC doped with 0.05% of Sudan Blue dye: (**a**) sketch of the NLC cell (*θ*_0_ = 30°) and optical fields coupling geometry with initial separation equal to 15 μm; (**b**) experimental verification of the co-propagation two orthogonally polarized light beams with optical powers corresponding to P_TM_ = 2 mW, P_TE_ = 2 mW, (**c**) P_TM_ = 6 mW, P_TE_ = 2 mW, (**d**) P_TM_ = 2 mW, P_TE_ = 4 mW, and (**e**) P_TM_ = 6 mW, P_TE_ = 4 mW. The dotted and dashed lines in (**b**–**e**) indicate the trajectories of the unperturbed and low-power TM and TE beams, respectively. (**f**) The trajectories of the co-propagated light beams correspond to (b,c): the TE beam in the unperturbed case (P_TE_ = 4 mW, black dashed line) and influenced by the TM beam of powers P_TM_ = 2 mW and P_TM_ = 6 mW (light and dark green solid lines, respectively), the TM beam in the unperturbed case (P_TM_ = 2 mW, black dotted line), and P_TM_ = 2 mW simultaneously propagated with P_TE_ = 4 mW (dark brown solid line); (**g**) the same as in (**f**) plotted for the TE beam in the unperturbed case (P_TE_ = 2 mW, black dashed line) and influenced by the TM beam of powers P_TM_ = 2 mW and P_TM_ = 6 mW (light and dark green solid lines), the TM beam in the unperturbed case (P_TM_ = 2 mW, black dotted line), and P_TM_ = 2 mW and P_TM_ = 6 mW simultaneously propagated with P_TE_ = 4 mW (light and dark brown solid lines, respectively).

The last part of our analysis explores the interaction of TE- and TM-polarized beams of the same wavelength, *λ* = 532 nm. The experimental setup, organized as schematically shown in Figure 5a, examines the propagation of TE and TM beams through an NLC cell, tracking the effects of polarization-dependent nonlinearities on beam behavior. The critical point here is that the reorientational nonlinearity is dominant over the TE beam’s thermal effect, thus ensuring spatial soliton’s existence. Conversely, thermal nonlinearity needed to be sufficiently strong to generate a thermal soliton for the TM beam, requiring a significant positive gradient in the ordinary refractive index. Similar to the above results, the TE beam, through its interaction with the optical axis, created a reorientational nematicon. The substantial absorption and resultant temperature gradient altered the TE beam’s trajectory by reducing the walk-off of the extraordinary beam. This modification in the walk-off angle corresponds to a displacement along the *y*-axis at a specific distance.

Interestingly, we observed that the extraordinary beam is repelled from the ordinary beam, yet the ordinary beam is simultaneously attracted to the extraordinary one. In this setup, the TE beam’s dual role of reorientation and induced positive temperature gradient (resulting in a negative refractive-index gradient for the TE beam and a positive one for the TM beam) was crucial. This negative refractive-index gradient affected the TE beam’s propagation direction, countering the TM beam’s repulsion. However, the TM beam’s repulsion proved to be stronger. At the same time, the TM beam experienced attraction towards the TE beam due to the positive temperature gradient relevant to the ordinary wave. The attractive and repulsive force ratio can be effectively controlled by varying the two input powers. In the Figure 5b,c, we can observe propagation of extraordinary beam of a power P_TE_ = 2 mW co-propagated with an ordinary wave of a power P_TM_ = 2 mW and P_TM_ = 6 mW, respectively. In such conditions, we can observe significant change in trajectory of a TE beam as a result of mutual interaction. For a higher power of TE beam, equal to P_TE_ = 4 mW, we can also observe a shift in the TM-polarized beam trajectory, towards TE beam, as shown in Figure 5d,e, for ordinarily polarized beams of a powers P_TM_ = 2 mW and P_TM_ = 6 mW.

The exact trajectories of the propagating beam that correspond to the cases presented in Figure 5b–e are plotted in Figure 5f,g. As can be clearly seen, the NLC medium, due to its peculiar polarization-dependent nonlinearity, both reorientation and thermal, via nonlinear interaction, can support the so-called diametric drive. Diametric drive means that the first beam must be drawn towards the second, while conversely, the second beam is repulsed by the first.

## 4. Conclusions

In conclusion, this intricate interdependence of thermal and reorientational effects, as governed by beam polarization and geometry, underscores the complex dynamics within doped NLCs, with profound implications for designing advanced photonic systems for controlling and manipulating light. The thermal and reorientational effects offer a deeper understanding of light–matter interactions in anisotropic media, paving the way for developing novel optical devices leveraging these solitonic properties.

The intricate dynamics discovered in our study, such as the diametric drive and interaction between TE- and TM-polarized beams, suggest several promising avenues for future research. Integration with photonic structures: The integration of NLC-based nonlinear phenomena with photonic crystals, waveguides, and microresonators can open new possibilities for on-chip optical signal processing, light routing, and switching. Such integration efforts can leverage the unique properties of NLCs for the development of reconfigurable photonic devices and circuits. Dynamic Control and Reconfigurability: Another promising direction involves developing methods for dynamic control of nonlinear optical phenomena in NLCs using external fields, temperature gradients, or light itself. This research could result in the creation of adaptive optical elements capable of real-time adjustment of their optical properties in response to changing environmental conditions or operational requirements.

The potential applications span a wide range of advanced photonic systems that exploit the unique properties of NLCs for the development of the next generation of optical devices. The intricate interplay between beam polarization, power, and the nonlinear properties of NLCs opens new avenues for designing advanced photonic devices. In particular, the ability to manipulate light propagation through reorientation and thermal nonlinearities in NLCs can be exploited for the design of tunable optical components, such as lenses, modulators and beam steering devices. These components have important applications in optical communications. They enhance signal processing capabilities and improve the efficiency of photonic circuits. In addition, the observed phenomena, such as the “diametric drive” effect, suggest innovative approaches to light control and manipulation in optical computing and information processing. Here, light beams can interact in unconventional ways to perform computational tasks or control signal paths. In addition, the sensitivity of NLCs to different wavelengths and polarization states can be exploited in sensors and imaging devices. This offers improved sensitivity tailored to specific environmental conditions. These applications highlight the potential of NLC-based photonic systems in signal processing, control, and switching.

## Data Availability

Data is contained within the article.
